# Supplementary material for: Network analysis of ballast-mediated species transfer reveals important introduction and dispersal patterns in the Arctic
Source: Sci Rep. 2020 Nov 11;10:19558. doi: 10.1038/s41598-020-76602-4 (PMC7658980; doi:10.1038/s41598-020-76602-4)
Supplement: Supplementary file 1 — Supplementary Information 1. [file 41598_2020_76602_MOESM1_ESM.docx]

Supplementary Materials for

**Network analysis of ballast-mediated species transfer reveals important introduction and dispersal patterns in the Arctic**

Mandana Saebi^1,2, +^, Jian Xu^1,3, +^, Salvatore R. Curasi^4, +^, Erin K. Grey^5^, Nitesh V. Chawla^1,2,^ David M. Lodge^6*^

^1^Department of Computer Science and Engineering, University of Notre Dame, Notre Dame, IN 46556, USA

^2^Center for Network and Data Science (CNDS), Notre Dame, IN 46556, USA

^3^ Citadel LLC, Chicago, IL 60603, USA

^4^Department of Biological Sciences, University of Notre Dame, Notre Dame, IN 46556, USA

^5^Division of Science, Mathematics and Technology, Governors State University, University Park, IL 60484, USA

^6^Cornell Atkinson Center for Sustainability, and Department of Ecology and Evolutionary Biology, Cornell University, Ithaca, NY 14850, USA

^*^Communicating author (dml356@cornell.edu)

+ The authors contributed equally to the manuscript.

**Supplementary Figures**

**
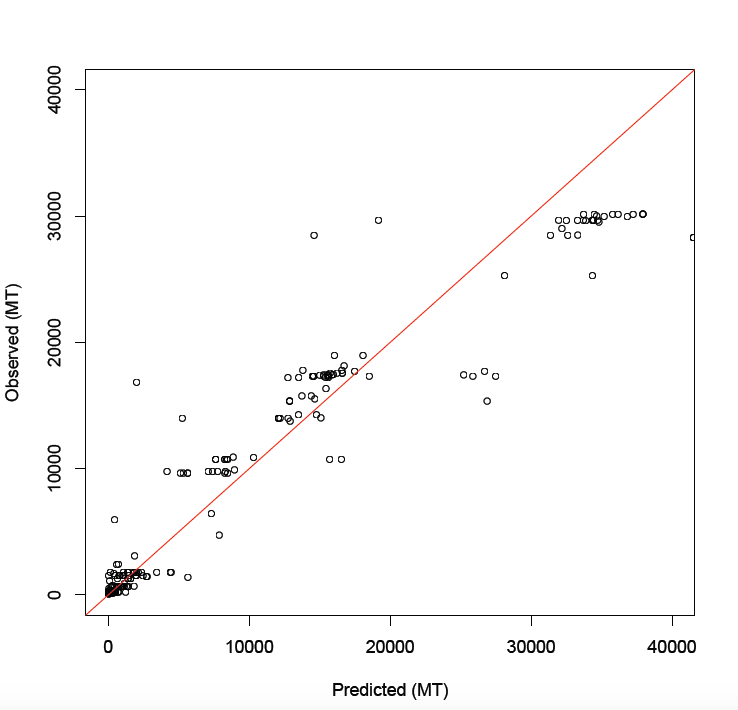
**

**Figure S1:** Observed ballast discharges versus predicted ballast discharges, showing prediction performance of the ballast discharge modeling. 1:1 regression line plotted for reference in red.

**
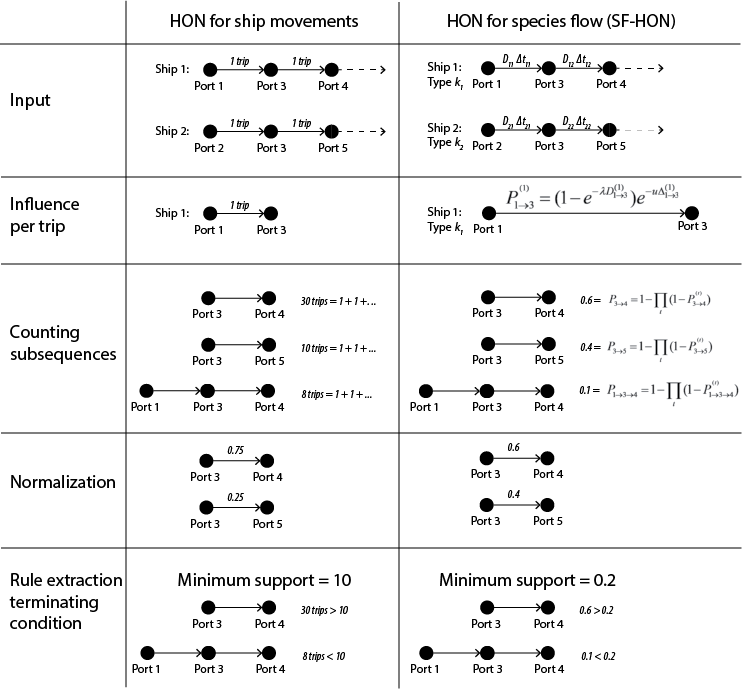
**

**Figure S2:** A comparison of the original HON construction algorithm that takes a single source of data (left) and the extended algorithm used in this work that can build SF-HON from multiple sources of data (right).

**Supplementary tables**

**Table S1**: Summary statistics and the evolution of shipping observed in the Lloyds Lists Intelligence data set.

|  |  | 1997-1998 | 1999-2000 | 2002-2003 | 2005-2006 | 2008-2009 | 2012-2013 | All years |
| --- | --- | --- | --- | --- | --- | --- | --- | --- |
| Introduction to the Arctic | Recipient ports | 104 | 112 | 102 | 97 | 88 | 78 | 183 |
|  | Direct pathways | 1,109 | 1,078 | 1,067 | 1,155 | 966 | 1,171 | 3,902 |
|  | Hub with the most incoming pathways | Murmansk  110 | Murmansk  117 | Murmansk  135 | Murmansk  167 | Murmansk  116 | Murmansk  158 | Murmansk  344 |
|  | Power-law distr. coeff. | -0.64 | -0.73 | -0.71 | -0.61 | -0.64 | -0.51 | -0.58 |
|  | Voyages | 3,522 | 3,136 | 3,633 | 4,676 | 3,877 | 5,454 | 24,298 |
|  | Sum DWT | 3.65×10^7^ | 3.21×10^7^ | 3.79×10^7^ | 5.70×10^7^ | 5.52×10^7^ | 7.07×10^7^ | 2.89×10^8^ |
|  | DWT per voyage | 1.04×10^4^ | 1.02×10^4^ | 1.04×10^4^ | 1.22×10^4^ | 1.42×10^4^ | 1.30×10^4^ | 1.19×10^4^ |
| Propagation in the Arctic | Recipient ports | 89 | 83 | 82 | 100 | 73 | 74 | 168 |
|  | Direct pathways | 414 | 318 | 354 | 348 | 304 | 522 | 1,269 |
|  | Hub with the most incoming pathways | Murmansk  18 | Murmansk  13 | Tromso  21 | Tromso  18 | Tromso  22 | Reykjavik  31 | Tromso  45 |
|  | Power-law distr. coeff. | -1.17 | -1.09 | -1.14 | -1.30 | -1.11 | -0.73 | -1.02 |
|  | Voyages | 2,579 | 1,856 | 3,271 | 3,246 | 3,404 | 9,710 | 24,066 |
|  | Sum DWT | 1.15×10^7^ | 8.93×10^6^ | 1.57×10^7^ | 2.00×10^7^ | 1.77×10^7^ | 5.75×10^7^ | 1.31×10^8^ |
|  | DWT per voyage | 4.47×10^3^ | 4.81×10^3^ | 4.79×10^3^ | 6.16×10^3^ | 5.21×10^3^ | 5.92×10^3^ | 5.46×10^3^ |

**Table S2**: Top ten potential species introduction and dispersal pathways ranked by $P_{i\to j}$ .

| Rank | Introduction pathway (non-Arctic → Arctic) | Dispersal pathway (Arctic → Arctic) |
| --- | --- | --- |
| 1 | Bremen, DEU → Narvik, NOR | Murmansk, RUS → Dudinka, RUS |
| 2 | Rotterdam, NLD → Murmansk, RUS | Dudinka, RUS → Murmansk, RUS |
| 3 | Hamburg, DEU → Narvik, NOR | Murmansk, RUS → Glomfjord, NOR |
| 4 | Rotterdam, NLD → Narvik, NOR | Kandalaksha, RUS → Murmansk, RUS |
| 5 | Hamburg, DEU → Murmansk, RUS | Hammerfest, NOR → Tromso, NOR |
| 6 | Amsterdam, NLD → Murmansk, RUS | Harstad, NOR → Tromso, NOR |
| 7 | Dunkirk, FRA → Narvik, NOR | Leirpollen, NOR → Grundartangi, ISL |
| 8 | Ymuiden, NLD → Narvik, NOR | Tromso, NOR → Hammerfest, NOR |
| 9 | Amsterdam, NLD → Narvik, NOR | Tromso, NOR → Bodo, NOR |
| 10 | Ghent, BEL →urmansk, RUS | Murmansk, RUS → Vitino, RUS |

**Table S3**: Top five introduction pathways for species in different environmental tolerance groups.

| Environmental tolerance groups | Introduction pathway |
| --- | --- |
| Salinity tolerance < 0.2 ppt  Temperature tolerance < 2.9 ℃ | Port Alfred, CAN → Churchill, CAN  Seaham, GBR → Akranes, ISL  Blyth, GBR → Straumsvik, ISL  Aberdeen, GBR → Straumsvik, ISL  Fraserburgh, GBR → Vestmannaeyjar, ISL |
| Salinity tolerance < 0.2 ppt  Temperature tolerance < 9.7 ℃ | Port Alfred, CAN → Churchill, CAN  Buckie, GBR → Eskifjordur, ISL  Seaham, GBR → Akranes, ISL  Cork, IRL → Glomfjord, NOR  Kirkwall, GBR → Reykjavik, ISL |
| Salinity tolerance < 2 ppt  Temperature tolerance < 2.9 ℃ | Tomakomai, JPN → Afognak, USA  Port Alfred, CAN → Churchill, CAN  Kushiro, JPN → Dutch Harbor, USA  Immingham, GBR → Vestmannaeyjar, ISL  Archangel, RUS → Seydhisfjordur, ISL |
| Salinity tolerance < 2 ppt  Temperature tolerance < 9.7 ℃ | Aughinish Island, IRL → Murmansk, RUS  Cape Town, ZAF → Straumsvik, ISL  Hunterston, GBR → Kirkenes, NOR  Immingham, GBR → Reykjavik, ISL  Port Talbot, GBR → Kirkenes, NOR |
| Salinity tolerance < 12 ppt  Temperature tolerance < 2.9 ℃ | Stravanger, NOR → Hafnarfjordur, ISL  Dalhousie, CAN → Kandalaksha, RUS  Haugesund, NOR → Hafnarfjordur, ISL  Tomakomai, JPN → Afognak, USA  Seven Islands, → CAN Grundartangi, ISL |
| Salinity tolerance < 12 ppt  Temperature tolerance < 9.7 ℃ | Rotterdam, NLD → Murmansk, RUS  Rotterdam, NLD → Narvik, NOR  Amsterdam, NLD → Murmansk, RUS  Amsterdam, NLD → Narvik, NOR  Ghent, BEL → Murmansk, RUS |

**Table S4**: Ports with the highest intra-Arctic introduction risks. Left: aggregated risk associated with single ship movements obtained in the conventional network. Right: indirect species flows through multiple steps of ship movements estimated using random walks on SF-HON.

| Rank | Risk of single-step direct introduction | Risk of multi-step indirect introduction |
| --- | --- | --- |
| 1 | Murmansk, RUS | Tromso, NOR |
| 2 | Tromso, NOR | Reykjavik, ISL |
| 3 | Dudinka, RUS | Murmansk, RUS |
| 4 | Glomfjord, NOR | Hammerfest, NOR |
| 5 | Hammerfest, NOR | Nuuk, GRL |
| 6 | Kirkenes, NOR | Kirkenes, NOR |
| 7 | Grundartangi, ISL | Harstad, NOR |
| 8 | Harstad, NOR | Dutch Harbor, USA |
| 9 | Hammerfall, NOR | Grundartangi, ISL |
| 10 | Bodo, NOR | Aasiaat, GRL |

**Table S5:** The fraction of non-zero releases (Z) calculated from the NBIC database organized by vessel type.

| Vessel type | Z |
| --- | --- |
| Bulker | 0.94 |
| Reefer | 0.38 |
| General cargo | 0.28 |
| Unknown | 0.23 |
| RoRo | 0.20 |
| Other | 0.14 |
| Tanker | 0.11 |
| Container | 0.06 |
| Passenger | 0.05 |
